# GlnR positively affects the acid resistance of *Lactiplantibacillus plantarum* from wine by regulating glutamate metabolism

**DOI:** 10.3389/fmicb.2025.1757806

**Published:** 2026-01-22

**Authors:** Ke Lu, Kan Shi, Yuxin Yuan, Yuanyuan Liu, Chuangyi Miao, Tao Pan, Pengfei Duan, Jangyong Wang, Shuwen Liu, Lili Zhao

**Affiliations:** 1 College of Enology, Northwest A&F University, Yangling, Shaanxi, China; 2 College of Economics and Management, Pu’er University, Puer, Yunnan, China

**Keywords:** acid resistance, GlnR, glutamate metabolism, *Lactiplantibacillus plantarum*, γ-aminobutyrate (GABA)

## Abstract

**Introduction:**

Owing to its remarkable capacity to modify the aroma profile of wine, *Lactiplantibacillus plantarum* (*L. plantarum*) derived from wine has emerged as a potential starter for malolactic fermentation. However, the inadequate acid resistance of this bacterium severely restricts its application. In some bacterial species, GlnR is considered a universal transcriptional regulator in response to acid stress.

**Methods:**

In this study, we determined the function of GlnR in the acid resistance of *L. plantarum* for the first time. RT-qPCR and yeast one-hybrid assays revealed a direct regulatory correlation between GlnR and genes associated with the glutamate metabolic pathway. Metabolomics analysis via liquid chromatography-mass spectrometry and fermentation studies confirmed that GlnR affected γ-aminobutyric acid (GABA) production.

**Results:**

The growth and survival rate of the knockout strain XJ25-Δ*glnR* were significantly lower than those of the wild-type strain XJ25. GlnR can directly bind to the promotor regions of the genes *glnA*, *gadB*, and *glms1*, thereby upregulating *gadB* transcription while downregulating *glnA* and *glms1* transcription, directing the increased metabolic flux toward GABA synthesis.

**Discussion:**

We present evidence that GlnR plays a vital role in the glutamate metabolic pathway and is a positive transcriptional regulator that can control the acid resistance of *L. plantarum* XJ25. Although GlnR interacts with *glnA*, *gadB*, and *glms1*, additional studies are warranted to determine how this interaction affects its acid resistance.

## 1 Introduction

Wine fermentation is a complex process, that includes alcoholic fermentation by yeast and malolactic fermentation (MLF) by lactic acid bacteria (LAB). MLF can improve the quality of wine and decrease its total acidity (Chen et al., 2022; Yang et al., 2022). This process becomes particularly significant in cold climate wine regions where its low pH (< 3.5) leading to MLF delay (Acevedo et al., 2020; Spano and Massa, 2006). Therefore, the ability of LAB to grow under acidic conditions is vital for its colonization in wine. If not, acids can exert some detrimental effects, including enzyme inactivation, DNA and protein damage, and LAB growth and metabolism modifications (Lyu et al., 2018). To mitigate acid stress, LAB employs various types of acid resistance mechanisms, including pH homeostasis maintenance, alkali production, metabolic regulation, exopolysaccharide production, and macromolecule repair (Mallick and Das, 2023). In general, *Lactiplantibacillus plantarum* (*L. plantarum*) is the major wine-associated LAB and can withstand the extreme conditions observed in wine production (Yang et al., 2022; Wang et al., 2020; Berbegal et al., 2016). At present, most studies have focused on the role of *L. plantarum* as an MLF starter culture for wine (Brizuela et al., 2019; Devi et al., 2022; Zhang et al., 2022), with research on acid resistance being scarce. Although the vital role of *mleA* in the acid stress response of *L. plantarum* WCFS1 has been investigated (Chen et al., 2022), the mechanisms underlying the acid resistance of *L. plantarum* remain unclear. Therefore, elucidating the mechanisms and patterns of microbial responses to acid resistance is vital for further applying *L. plantarum* XJ25 in wine.

GlnR is a universal transcriptional regulator of nitrogen metabolism in some bacteria. It can be divided into two types: MerR-type GlnR, including those in the family LAB and *Bacillaceae*, and OmpR-type GlnR, including those in the family *Actinomycetaceae* (Yao et al., 2014; Randazzo et al., 2017). In *Streptococcus mutans*, GlnR can inhibit the expression of the *citBZC* operon under low pH conditions. *citBZC* downregulation improves citrate to pyruvate conversion with H^+^ consumption. Therefore, *glnR* knockout decreased the cell survival capability of *Streptococcus mutans* (Chen et al., 2010). On the other hand, in *Streptococcus salivarius*, GlnR can activate the expression of the *ure* operon to elevate acid resistance under low pH conditions (Huang and Chen, 2016). In *L. brevis*, with the presence of 10 mM sodium glutamate at pH 2.8, the survival colony number of the wild-type strain was significantly lower than that of the *glnR* knockout strain. After 3 h, the survival colony number of the *glnR* knockout and wild-type strains was 2.6 × 10^9^ CFU/mL and 1.1 × 10^9^ CFU/mL, respectively, suggesting that *glnR* negatively regulates acid resistance (Gong et al., 2020). Therefore, GlnR can function as an activator or inhibitor in response to acid resistance at least in these three strains. Presumably, GlnR modulates acid resistance in LAB via diverse regulatory mechanisms. However, the contribution of GlnR to the acid resistance of *L. plantarum* remains undetermined experimentally.

The glutamate decarboxylase (GAD) system is an important defense mechanism against acid damage in LAB (Paudyal et al., 2020; Yang et al., 2021; Gong et al., 2019). It can catalyze glutamate decarboxylation, thereby producing γ-aminobutyric acid (GABA). Owing to the exchange between glutamate and GABA, protons are consumed inside the cells. Therefore, the intracellular pH increases, thereby protecting the cell from acid stress (Xu et al., 2023a; Wu et al., 2017; Ma et al., 2012). Furthermore, many researchers have reported that exogenous amino acids may enhance the GAD acid resistance of bacteria to survive under acidic conditions (Paudyal et al., 2020; Muchaamba et al., 2019; Park and Diez-Gonzalez, 2004). Yin et al. (2017) have reported that 0.5 g/L exogenous glutamate and aspartate can enhance the acid resistance of *Acetobacter pasteurianus* by promoting the synthesis of fatty acids, nucleic acids, and glutathione to regulate intracellular pH. Furthermore, Xu et al. (2023b) have reported that glutamate, lysine, and arginine can protect bacterial cells from damage caused by acid stress by increasing intracellular pH and ATP levels. Senouci-Rezkallah et al. (2011) have reported that glutamate in the culture condition plays a vital role in regulating intracellular pH and enhancing acid resistance under acid stress conditions in *Bacillus cereus*. Another study has revealed that approximately 30% and 25% of *Escherichia coli* survived in the presence of only glutamate and glutamine as the amino acid supplement at pH 2.5 (Lu et al., 2013). A literature review has revealed that studies on the effect of exogenous glutamate on the acid stress responses of *L. plantarum* are limited. Moreover, the GAD system is present in *L. plantarum* and has only one GAD-coding enzyme, g*adB* (Wu et al., 2017; Su et al., 2011). However, whether GlnR encodes glutamate metabolism to protect *L. plantarum* against the deleterious effects of acids remains unclear.

In this study, we investigated the contribution of the transcriptional regulator GlnR to the acid resistance of *L. plantarum* XJ25. For this, *glnR* knockout and overexpression strains were constructed, followed by cell survival assays, *glnR* relative expression analysis, and L-malic acid concentration measurements. RT-qPCR and yeast one-hybrid assays were performed to study the interaction between GlnR and multiple genes associated with glutamate metabolism. Furthermore, metabolomics analysis via liquid chromatography (LC)-mass spectrometry (MS) was performed to identify the differentially accumulated metabolites (DAMs) between XJ25 and XJ25-Δ*glnR*. Our study findings can substantially enrich our understanding of the molecular regulatory mechanisms underlying the acid stress response governed by GlnR.

## 2 Materials and methods

### 2.1 Strains, plasmids, and culture conditions

The knockout and expression plasmids were maintained in *Escherichia coli* DH5a (Shanghai Weidi Biotechnology Co., Ltd, China), and grown at 200 rpm on Lysogeny broth (LB). The *L. plantarum* XJ25 were cultured at 37 °C in de Man Rogosa and Sharpe (MRS) medium (Meng et al., 2021). The Y187 yeast strain were cultured at 30 °C on SD/-Leu/-Trp (PM2222, Beijing Coolaber Co., Ltd, China). The experiments were performed in minimum CDM medium, which containing 6.7 g/L YNB (PM2070, Beijing Coolaber Co., Ltd, China), 10 g/L glucose, 5 kinds of nucleotide bases (10 mg/L), sodium glutamate (Glu) was supplemented at a final concentration of 10 mM if necessary, and pH 3.2 adjusted with HCl. The strains XJ25 and XJ25-Δ*glnR* were incubated in CDMA media (Terrade et al., 2009) to analysis of the metabolites. The pHis2 and pGADT7 plasmids were applied in yeast one-hybrid assays (Beijing Coolaber Co., Ltd, China). Appropriate antibiotics were added to the medium when needed: 50 μg/mL kanamycin for pHis2 plasmid and *E. coli*, 50 μg/mL erythromycin for *L. plantarum*, 100 μg/mL ampicillin for pGADT7 plasmid.

All bacterial strains and plasmids constructed and used in this study were listed in Supplementary Table 1. Primers used in this study were listed in Supplementary Table 2. Restriction enzymes were purchased from Takara [Bao Biological Engineering (Dalian) Co., Ltd, Dongbei, China].

### 2.2 Construction of glnR plasmids and strains ^宝生物工程(大连)有限公司^

#### 2.2.1 Construction of glnR knockout and expression plasmids

Plasmid pLCNICK derived from our laboratory was used as a starting point and was linearised by double digestion with XbaI and ApaI (Huang et al., 2019). Two fragments flanking, *glnR*-up and *glnR*-down were amplified from *L. plantarum* XJ25 strain using the primers *glnR*-up-1/*glnR*-up-2 and *glnR*-down-1/*glnR*-down-2, respectively. A sgRNA framework that targets *glnR*-sgRNA was obtained using the primers sgRNA-1/*glnR*-sgRNA-2 with pLCNICK as the template. These three fragments were then assembled with *glnR*-up-1 and *glnR*-sgRNA-2 by overlap extension PCR. Subsequently, the backbone of pLCNICK and previous three fragment were assembled by NovoRec plus One step PCR Cloning Kit (05302309, Novoprotein, China) to produce a new plasmid, pLCNICK-Δ*glnR*. Positive clones were detected using the primers pLCNICK-text-1 and pLCNICK-text-2.

Plasmid pMG36ek11 derived from Yang et al. (2022) was used as a starting point and linearized by PCR amplification. Two fragments are connected to form plasmid pMG36ek11-glnR, the backbone of pMG36ek11 (amplified using primers P11-2 and pMG36e-express-3), the glnR fragment (amplified using glnR-express-1 and glnR-express-2 with *L. plantarum* XJ25 strain as the template).

#### 2.2.2 Transformation

Heat shock transformation was carried out according to the instructions of *Escherichia coli* DH5a. After shaking (pLCNICK-ΔglnR at 30 °C, 36ek11-glnR at 37 °C), these plasmids were delivered into *L. plantarum* XJ25 by electroporation as previously described (Huang et al., 2019; Wang et al., 2021). Positive clones were verified by PCR amplification with the primers glnR-in-1 and glnR-in-2, or glnR-ha-1 and glnR-hp-2 to obtain the glnR knockout mutants. Similarity, the primers pMG36e-test-1 and pMG36e-test-2 were used to verify glnR overexpression mutants. PCR products were sequenced to confirm the knockouts and expressions (Yangling Aoke Biotech Co. Ltd, Shaanxi, China).

#### 2.2.3 Identification

The length of the *glnR* knockout plasmid correlated with the expected size of 14,365 bp. To construct the *glnR* knockout strain, the verified plasmid was transformed into *L. plantarum* XJ25. Two *glnR* knockout mutants (bands 1 and 3), called XJ25-Δ*glnR*, were achieved (Supplementary Figure 1A); these strains were evidently distinguishable from the wild-type strains (bands 2, 4–23). Furthermore, sequencing of the PCR amplicon confirmed the precise knockout of *glnR* (Supplementary Figure 1B). The length of the *glnR* overexpression plasmid (pMG36ek11-*glnR*) correlated with the expected size of 4,574 bp. This verified *glnR* overexpression plasmid was transformed into *L. plantarum* XJ25-Δ*glnR* to construct XJ25-Δ*glnR*-36ek11 and XJ25-Δ*glnR*-36ek11-glnR. As demonstrated in Supplementary Figure 1C, the strain exhibited the expected electrophoretic bands (band 4), as verified by PCR, and was evidently distinguishable from XJ25-ΔglnR-36ek11 (band 3). This indicates the successful expression of glnR in XJ25-ΔglnR.

### 2.3 Acid resistance

To evaluate acid resistance, XJ25 and XJ25-ΔglnR were grown on MRS liquid medium at appropriate pH points (6.0, 3.8, 3.6, 3.2). The early exponential phase (OD_600_ value of 0.3) was applied and inoculated 1% into MRS liquid medium, followed by culturing for 40 h at 37 °C. Cell growth was monitored by a multi-functional microplate detector (Spectra Max M2, BioTek Instrument Co., Ltd, USA). Difference in pH units between the initial and final pH values of XJ25 and XJ25-ΔglnR after cultivation at 37 °C for 48 h were detected by pH meter (PB-10, Sartorius Co., Germany). To determine the cell survival, the previously exponential phase was washed three times with 0.85% sodium chloride. Then added into CDM liquid medium with pH 3.2 separately, followed by culturing for 12 h at 37 °C (the time periods of acid stress were 12, 9, 6, 3, and 0 h). All samples were immediately diluted and spread on MRS agar plates. In addition, 5 μL of dilutions was dripped on MRS agar plates, and the plates were photographed.

### 2.4 Cell surface properties

XJ25 and XJ25-ΔglnR cell morphology were detected using SEM (JSM-6360LV, Japan). Samples were handled according to the method of Yang et al. (2019). Briefly, XJ25 and XJ25-ΔglnR were grown in MRS liquid medium at appropriate pH points 3.2 for 48 h. Cell suspension was fixed with 2.5% (v/v) glutaraldehyde for 12 h at 4 °C and washed four times with phosphate buffered saline (pH 6.8). Next, samples were dehydrated with a graded series of ethanol solutions [10, 30, 50, 70, 80, and 90% (v/v)] every 15–20 min in sequence, then dehydrated with 100% ethanol solutions every 30 min for three times. Finally, samples were dried for 4 h in a critical point dryer, sputter-coated with gold and watched by SEM.

### 2.5 Evaluation of metabolism concentration at the process of MLF by Y15

The strains XJ25, XJ25-Δ*glnR*, XJ25-Δ*glnR*-36ek11, and XJ25-Δ*glnR*-36ek11-glnR were incubated, when OD_600_ reached 1.0, the 5% (v/v) inoculum of the overnight culture was transferred into fresh CDMm medium containing 3 g/L L-malic acid, incubated at 37 °C for 24 h. Samples were collected at appropriate time points. L-malic acid, L-lactic acid, glucose and D-lactic acid were determined by enzymatic kit analysis according to the manufacturer’s recommendations with some modifications. In brief, 1 mL of each sample was collected at different fermentation time, incubated the mix for 15 min in a water bath (80 °C) to stop enzymatic reactions, centrifuged at 12,000 *r* for 2 min and then collected the supernatant and used the filtrate for the assay, diluted if necessary. Standards and reagents are provided ready to use in Kit (12800-12803, BioSystems, Barcelona, Spain). Components are stable and stored at −4 °C until the expiry date stated in the label during their use. An automatic analyzers Y15 Biosystems (Barcelona, Spain) is supplied with easy-to-use software to facilitate laboratory routine. The metabolisms test is already programmed in the software. All parameters are shown in different tabs and there is no need to change any parameter (Tobeña et al., 2021).

### 2.6 RT-qPCR

The strains XJ25, XJ25-Δ*glnR*, XJ25-Δ*glnR*-36ek11, and XJ25-Δ*glnR*-36ek11-glnR were applied. Cells were collected by the same method as 2.5, and then resuspended in the same volume of fresh CDMm medium, cultured for 6 h. Total RNA was extracted with the miRNEasy kit (DP424, Tiangen Biochemical Technology Co., Ltd, China) following the manufacturer’s protocol. The qualified RNA was stored at −80 °C. The mRNA was extracted and reverse transcribed using an Evo M-MLV RT Mix Kit [A4A2930, Accurate Biotechnology (Hunan) Co., Ltd, China]. The abundance of mRNAs coding for glnR, gabD, gdh, gadB, purQ, glnA, glms1, purF, lp-0433 and carB (adjacent genes in the genome), and 16S rRNA (reference gene) were measured by amplifying the genes using corresponding cDNAs as PCR templates. The microRNA expression was applied with SYBR Green Premix Pro Tap HS qPCR Kit [A4A3045, Accurate Biotechnology (Hunan) Co., Ltd, China] following the manufacturer’s protocol and PCR was performed on the RT-qPCR instrument (CFX-Opus96, Bio-Rad, USA). The relative gene expression was obtained using method by Meng et al. (2021).

### 2.7 Yeast one-hybrid assay

A 100∼500 bp sequence of the glnA (verified by the primers His-glnA-express-1 and His-glnA-express-2), gadB (verified by the primers His-gadB-express-1 and His-gadB-express-2), glms1 (verified by the primers His-glms1-express-1 and His-glms1-express-2) and purQ (verified by the primers His-purQ-express-1 and His-purQ-express-2) promoters were inserted into the SacI and EcoRI restriction sites of the pHis2 plasmid. Next, the 381 bp glnR (verified by the primers AD-glnR-express-1 and AD-glnR-express-2) sequences were cloned into the NdeI and BamHI restriction sites of the pGADT7 plasmid. The constructed prey and bait plasmids were co-transformed into yeast strain Y187 according to the manufacturer’s recommendations (YH1011, Beijing Coolaber Co., Ltd, China). PGADT7-53, together with pHis2-p53 constructions serving as the positive control, were all transformed into Y187, and the growth of the yeast cells were monitored on a synthetic media (SD/-Leu/-Trp) for 2–3 d. The successful co-transformants were resuspended in 1 mL of 0.9% NaCl sterile water, with OD_600_ adjusted to 0.2 and then diluted (i.e., OD_600_ = 0.2, 0.02, 0.002, 0.0002). Finally, 10 uL of each dilution was placed successively on the SD/-Leu/-Trp, SD/-Leu/-Trp/-His screening plates supplied with specific concentrations of 3-amino-1,2,4-triazole (3AT) and incubated at 30 °C for 3 d to test the interactions between bait and prey proteins (He et al., 2023).

### 2.8 LC-MS analysis

To further investigate the effect of glnR on *L. plantarum* XJ25 metabolites, the strains XJ25 and XJ25-ΔglnR were incubated in CDMA media, a new chemically defined medium for wine LAB. All samples were acquired using the instrumental parameters of the LC-MS system. HPLC was performed using the Waters ACQUITY UPLC HSS T3 column (1.8 μm, 2.1 mm × 100 mm). The solvent system comprised water with 0.1% formic acid (solvent A) and acetonitrile with 0.1% formic acid (solvent B). The mobile phase gradient program was as follows: starting with 95% A and 5% B, transitioning to 35% A and 65% B within 5 min, linear gradient to 1% A and 99% B within 1 min, and maintained under this condition for 1.5 min. Subsequently, a composition of 95% A and 5% B was adjusted within 0.1 min and maintained for 2.4 min. The flow velocity was 0.40 mL/min, the column oven temperature was 40 °C, and the injection volume was 4 μL. Data were acquired in the information-dependent acquisition mode using Analyst TF 1.7.1 Software (Sciex, Concord, ON, Canada). The source parameters were as follows: ion source gas I, gas II, and curtain gas were set at 50, 60, and 35 psi, respectively; temperature, 550 °C; declustering potential, 80 V (positive ion mode)/−80 V (negative ion mode), respectively; and ion spray voltage floating, 5,500 V (positive ion mode)/−4,500 V (negative ion mode); The TOF MS scan parameters were set as follows: mass range, 50–1,250 Da; accumulation time, 200 ms; and dynamic background subtract, on. An ELISA Kit (GABA-1-W, Suzhou Comin Co., Ltd, China) was used according to the manufacturer’s recommendations to measure GABA levels. Metabolites were quantified using multiple reaction monitoring. Metabolites with VIP ≥ 1, and FC ≤ 0.83 or ≥ 1.2 were selected as the differential compounds.

### 2.9 Preparation of the model wine

Model wine simulates the physicochemical properties of real wine was used to complete testing and reduce experimental costs. The model wine formula is as follows: grape juice 10 g/L, glucose 2 g/L, fructose 2 g/L, NaCl 0.2 g/L, NH_4_SO_4_ 1 g/L, KH_2_PO_4_ 2 g/L, yeast extract 4 g/L, malic acid 3 g/L, MnSO_4_ 0.05 g/L, MgSO_4_ 0.2 g/L, sodium glutamate 2 g/L, 12% (v/v) ethanol, and pH 3.6 adjusted with HCl. The strains XJ25 and XJ25-ΔglnR were incubated in MRS media, when OD_600_ reached 1.5, washed by 0.85% sodium chloride, than the 1% (v/v) inoculum was transferred into the model wine, fermented at 20 °C for 8 days. The survival colony number, the concentration of L-malic acid and GABA were determined.

### 2.10 Statistical analysis

The data were the average values of three replicates (metabolites were detected in six replicates). One-way ANOVA followed by Duncan’s multiple-range test was conducted to test the significant differences (*P* < 0.05) between the means. The statistical software utilized was SPSS 25.0 (SPSS, Chicago, IL, USA). The experimental results were plotted by GraphPad Prism. The software tools SnapGene 3.2.1 and HDOCKlite were used to analyze the gene sequence.

## 3 Results

### 3.1 Construction of glnR mutant strains

The length of the glnR knockout plasmid correlated with the expected size of 14,365 bp. To construct the glnR knockout strain, the verified plasmid was transformed into *L. plantarum* XJ25. Two glnR knockout mutants (bands 1 and 3), called XJ25-ΔglnR, were achieved (Supplementary Figure 1A); these strains were evidently distinguishable from the wild-type strains (bands 2, 4–23). Furthermore, sequencing of the PCR amplicon confirmed the precise knockout of glnR (Supplementary Figure 1B). The length of the glnR overexpression plasmid (pMG36ek11-glnR) correlated with the expected size of 4,574 bp. This verified glnR overexpression plasmid was transformed into *L. plantarum* XJ25-ΔglnR to construct XJ25-ΔglnR-36ek11 and XJ25-ΔglnR-36ek11-glnR. As demonstrated in Supplementary Figure 1C, the strain exhibited the expected electrophoretic bands (band 4), as verified by PCR, and was evidently distinguishable from XJ25-ΔglnR-36ek11 (band 3). This indicates the successful expression of glnR in XJ25-ΔglnR.

### 3.2 GlnR positively regulates the acid resistance of L. plantarum XJ25

**FIGURE 1:**
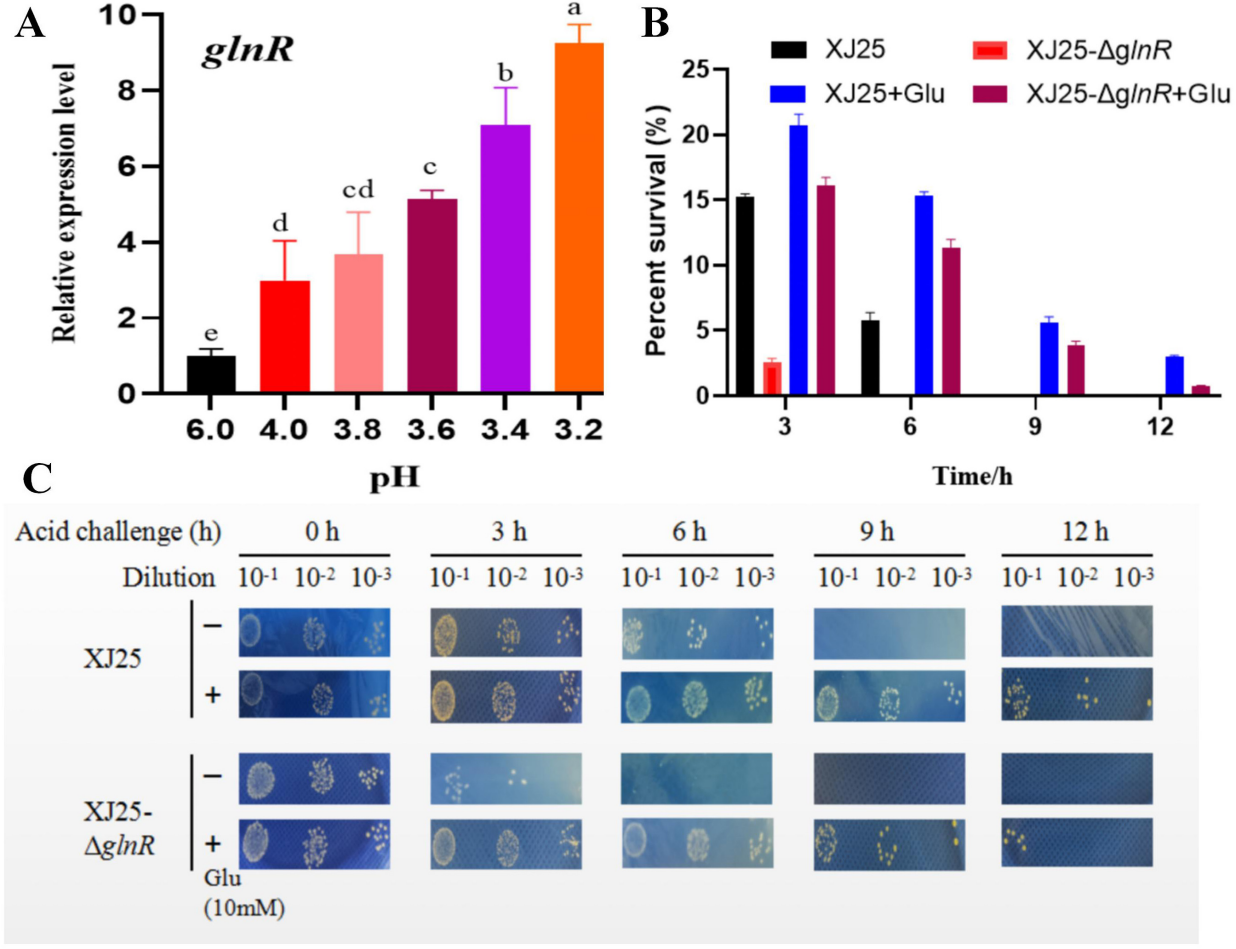
Acid tolerance of the wild-type (XJ25) strain and *glnR* deletion strain (XJ25-Δ*glnR*). **(A)** The expression level of *glnR* in XJ25 under distinct pH condition; **(B)** Percent survival of strain XJ25 and strain XJ25-Δ*glnR* in CDM medium (pH 3.2) with Glu (+) or without Glu (–); **(C)** cell viability of strain XJ25 and strain XJ25-Δ*glnR* in CDM medium (pH 3.2) with Glu (+) or without Glu (–). The images represent serial dilutions of the cultures in tenfold steps. Acid tolerance were detected in three replicates. Letters a–e indicate statistically significant differences (*P* < 0.05).

To determine the contribution of *glnR* to acid resistance, *glnR* expression was measured in XJ25 under different pH conditions (Figure 1A). We observed that relative *glnR* expression was considerably higher in the batches with lower pH values than in the batch at pH 6.0. The highest relative *glnR* expression was observed in the batch at pH 3.2 (9.250.40), suggesting that *glnR* positively regulates the acid resistance of *L. plantarum* XJ25.

In CDM with or without sodium glutamate, the survival rate of the wild-type strain was significantly higher than that of XJ25-Δ*glnR* (Figure 1B). After 3 h of acid stress, the survival rate of XJ25 was 15.1%; however, it was 2.5% for XJ25-Δ*glnR*. Similarly, the survival rate of XJ25+Glu was higher than that of XJ25-Δ*glnR*+Glu (20.7% vs. 16.1%). During acid stress for 6 h, the survival rate of XJ25 (15.3%) in Glu-supplemented CDM was the most resistant, whereas that of XJ25-Δ*glnR* in only CDM was more sensitive. These results suggest that XJ25-Δ*glnR* is more sensitive to acid stress than the wild-type XJ25 strain. Interestingly, after 9 and 12 h of stress, both XJ25 and XJ25-Δ*glnR* were sensitive to acid stress in the absence of Glu. Therefore, cell survival under acid stress further suggests that *glnR* and Glu are vital for the acid resistance of *L. plantarum* (Figure 1C).

**FIGURE 2:**
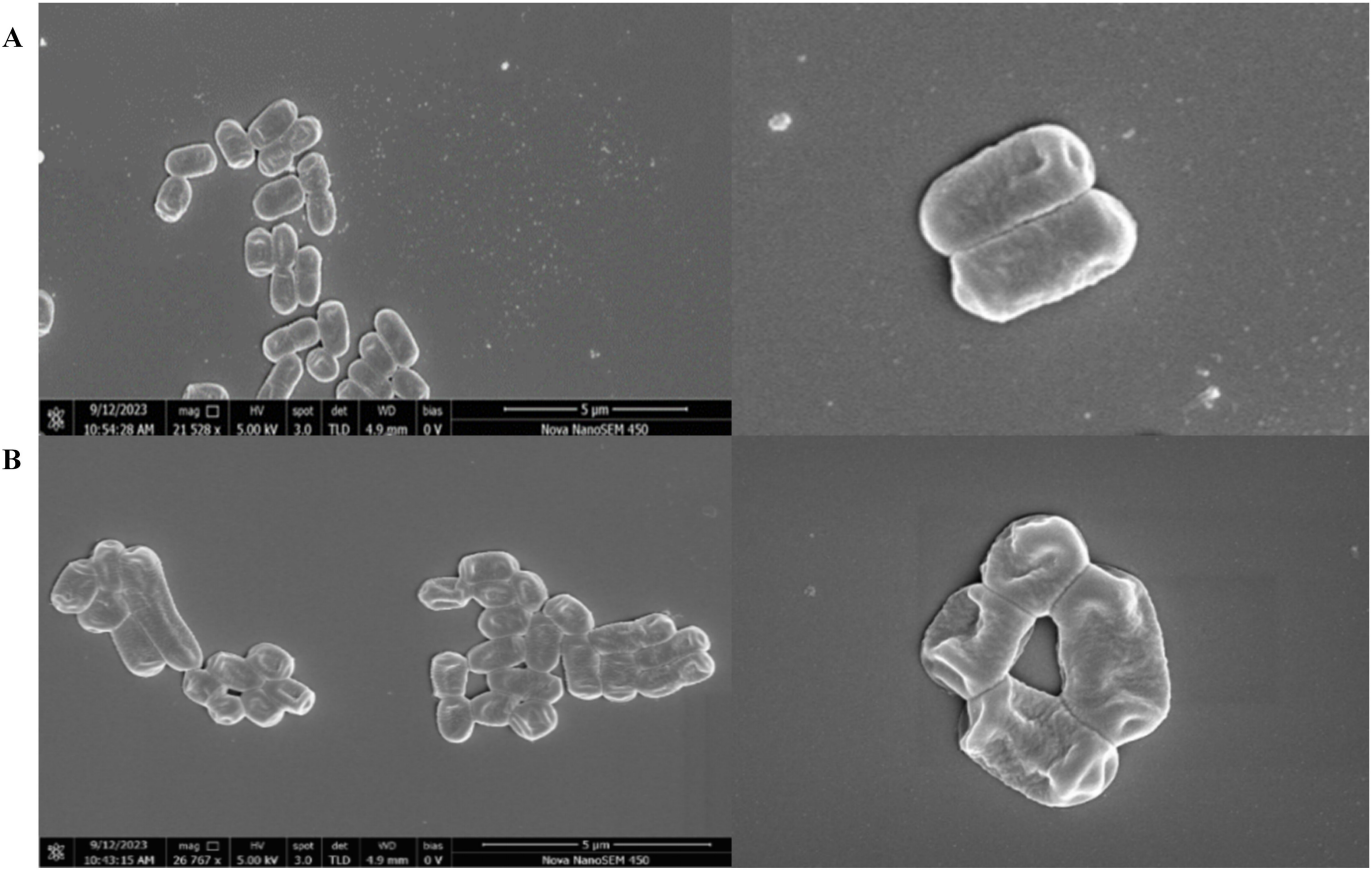
SEM of strain XJ25 **(A)** and strain XJ25-Δ*glnR* **(B)** in MRS medium with pH 3.2.

SEM was performed to assess the cell surface characteristics of XJ25 and XJ25-ΔglnR at pH 3.2. Some differences in morphological properties and surface were observed under acid stress. XJ25-ΔglnR cells exhibited obvious wrinkling and collapse (Figure 2A); in contrast, XJ25 cells were smoother, fuller, and more complete (Figure 2B).

### 3.3 GlnR can directly regulate MLF in CDMm

**FIGURE 3:**
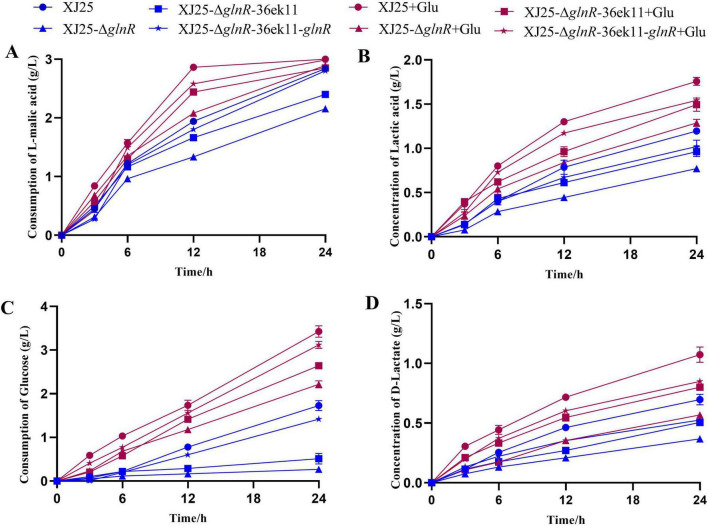
Changes in metabolite concentration in the CDMm medium with the wild-type and the mutants. **(A)** L-malic acid utilization; **(B)** L-lactic acid production; **(C)** Glucose utilization; **(D)** D-lactic acid production. Data are expressed as mean ± standard deviation (*n* = 3).

Subsequently, to elucidate the importance of glnR during MLF, all strains were cultured in CDMm. Figure 3 illustrates the changes in L-malic acid, L-lactic acid, glucose, and D-lactic acid concentrations during 24 h. Most of the L-malic acid present in the culture medium (0.14 g/L residual L-malic acid) was utilized by wild-type XJ25+Glu after 12 h of culture (Figure 3A). However, the L-malic acid present in the medium of other strains was not utilized after 24 h of culture. Furthermore, except for XJ25-ΔglnR and XJ25-ΔglnR-36ek11, most strains could entirely use L-malic acid after 24 h of culture in the absence of Glu. The residual L-malic acid concentration in the culture medium of the two glnR knockout mutants, namely, XJ25-ΔglnR (0.21 g/L) and XJ25-ΔglnR-pMG36ek11 (0.79 g/L), was much higher than that in the culture medium of XJ25; this suggests that XJ25 glnR knockout mutants could not entirely use L-malic acid without the help of Glu. For L-lactic acid production (Figure 3B), after 24 h of culture, no obvious differences were observed in L-lactic acid concentration in XJ25-ΔglnR-pMG36ek11, XJ25-ΔglnR-36ek11-glnR, XJ25-ΔglnR-pMG36ek11+Glu, and XJ25-ΔglnR-36ek11-glnR+Glu. The lowest L-lactic acid concentration was observed in XJ25-ΔglnR (0.77 g/L), whereas the highest concentration was observed in XJ25+Glu (1.76 g/L). The L-lactic acid concentration in XJ25-ΔglnR was 2.29-fold lower than that in XJ25+Glu. For glucose (Figure 3C), we observed that glucose utilization was considerably higher in the strains with Glu addition than in those without Glu addition. After 12 h of culture, the glucose concentration in the culture medium of two glnR knockout mutants, namely, XJ25-ΔglnR (0.27 g/L) and XJ25-ΔglnR-pMG36ek11 (0.51 g/L), was considerably lower than that in the culture medium of XJ25 (1.73 g/L). Furthermore, the glucose utilization rate of XJ25 was 6.41 times higher than that of XJ25-ΔglnR. Moreover, similar trends were observed in the strains with Glu addition, primarily owing to glnR inactivation, weakening glucose catabolism. For D-lactic acid production (Figure 3D), at 24 h, D-lactic acid concentration was up to 1.07 g/L in XJ25+Glu; this was 45.8% higher than that in XJ25-ΔglnR+Glu. Furthermore, D-lactic acid concentration in XJ25 (0.70 g/L) was higher than that in XJ25-ΔglnR (0.37 g/L).

### 3.4 Transcriptional regulation of glutamate metabolism genes by glnR via RT-qPCR analysis

**FIGURE 4:**
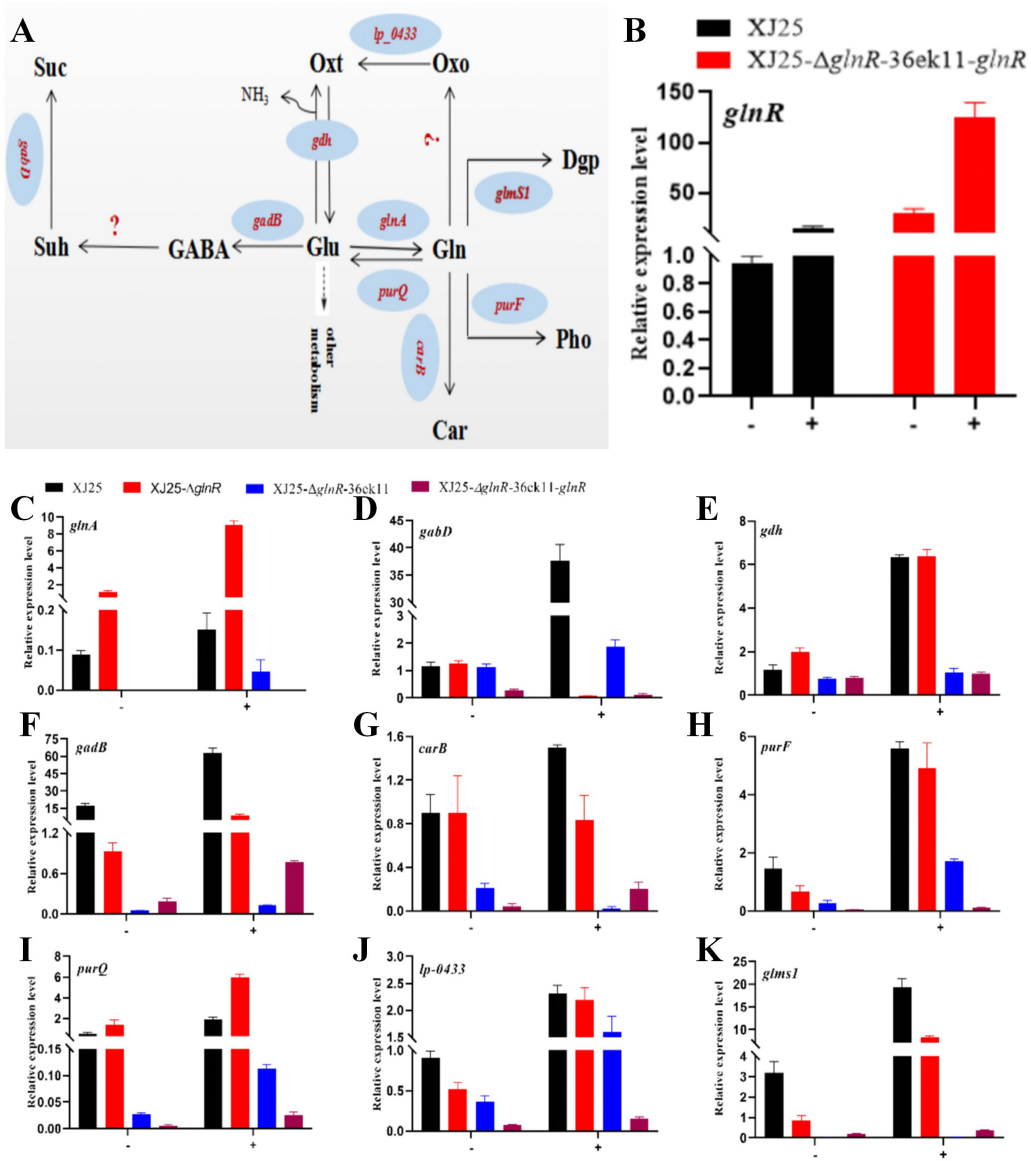
Role of *glnR* in controlling expression of glutamate metabolism genes in the wild-type and the mutants. **(A)** Glutamate biosynthesis and metabolic pathway; **(B)** Expression levels of *glnR* in XJ25 and XJ25-Δ*glnR*-36ek11-*glnR*; Expression levels of *glnA* **(C)**, *gabD* **(D)**, *gdh* **(E)**, *gadB* **(F)**, *carB* **(G)**, *purF* **(H)**, *purQ* **(I)**, *lp-1433* **(J)**, *glms1* **(K)** in *L. plantarum* XJ25, XJ25-Δ*glnR*, XJ25-Δ*glnR*-36ek11, and XJ25-Δ*glnR*-36ek11-*glnR*. Data are expressed as mean ± standard deviation (*n* = 3). Glu, glutamate; Gln, glutamine; GABA, γ-aminobutyrate; Suh, succinate semialdehyde; Suc, succinate; Oxt, 2-oxoglutarate; Oxo, 2-oxoglutaramate; Car, carbamoyl phosphate; Dgp, D-glucosamine 6-phosphate; Pho, 5-phosphoribosylamine. The question mark indicated that the gene required for the biochemical reaction was not found in the genome of *L. plantarum* XJ25.

To elucidate the regulatory mechanism of *glnR* on glutamate metabolism-mediated acid resistance, we examined the expression of the genes associated with glutamate biosynthesis and metabolic pathways, including *glnR*, glutamine synthetase-encoding gene (*glnA*), *gabD*, *gdh*, *gadB*, *carB*, *purF*, *purQ*, *lp-0433*, and glucosamine-6-phosphate synthase (*glms1*) (Figure 4A). Figure 4B illustrates that the relative expression of *glnR* was significantly higher in XJ25-Δ*glnR*-36ek11-*glnR* and XJ25-Δ*glnR*-36ek11-*glnR*+Glu than in wild-type XJ25. Compared with XJ25, the expression increased 16. 4-, 30. 2-, and 124.4-fold in XJ25+Glu, XJ25-Δ*glnR*-36ek11-*glnR*, and XJ25-Δ*glnR*-36ek11-*glnR*+Glu, respectively; this suggests that the pMG36ek11 plasmid possesses a high expression efficiency and that Glu can upregulate *glnR* expression to enhance its acid resistance. For *glnA* (glutamine synthetase) (Figure 4C), compared with XJ25, a 12.16-fold increase in expression was observed in XJ25-Δ*glnR*, whereas a 9.05-fold increase was observed in XJ25+Glu; this indicates that *glnR* can negatively regulate *glnA* transcription. For *gabD* (Figure 4D), no evident differences were observed in XJ25 and XJ25-Δ*glnR*, with the highest expression in XJ25+Glu. For *gdh* (glutamate synthetase) (Figure 4E), the expression was slightly higher in XJ25-Δ*glnR* than in XJ25; however, no evident differences were observed between XJ25+Glu and XJ25-Δ*glnR*+Glu; similar results were observed for XJ25-Δ*glnR*-36ek11, XJ25-Δ*glnR*-36ek11-*glnR*, XJ25-Δ*glnR*-36ek11+Glu, and XJ25-Δ*glnR*-36ek11-*glnR*+Glu. For *gadB* (GAD) (Figure 4F), compared with the XJ25, a 17.5- and 0.5-fold decrease in expression was observed in XJ25-Δ*glnR* and XJ25-Δ*glnR*+Glu and a 3.61-fold increase in expression was observed in XJ25+Glu, respectively. Furthermore, the expression was considerably higher in XJ25-Δ*glnR*-36ek11-*glnR* and XJ25-Δ*glnR*-36ek11-*glnR*+Glu than in the corresponding strains XJ25-Δ*glnR*-36ek11 and XJ25-Δ*glnR*-36ek11+Glu, respectively; this indicates that *glnR* positively regulates *gadB* transcription. For *carB* (Figure 4G), no evident differences were observed between XJ25 and XJ25-Δ*glnR*; however, the expression in XJ25-Δ*glnR*+Glu was 1.73-fold lower than that in XJ25+Glu. For *purF* (Figure 4H) and *lp-1433* (Figure 4J), their expression was slightly higher in XJ25 than in XJ25-Δ*glnR*; this is contradictory to the expression in XJ25-Δ*glnR*-36ek11 and XJ25-Δ*glnR*-36ek11-*glnR*; nevertheless, no evident differences were observed between XJ25+Glu and XJ25-Δ*glnR*+Glu, indicating that the genes *purF* and *lp-1433* were not affected by *glnR*. In the presence and absence of Glu, the expression of *purQ* (Figure 4I) was slightly lower in XJ25 than in XJ25-Δ*glnR*. Compared with XJ25, a 2. 9-, 3. 9-, 12.2-fold increase in expression was observed in XJ25-Δ*glnR*, XJ25+Glu, and XJ25-Δ*glnR*+Glu, respectively. Moreover, in the presence and absence of Glu, *glms1* expression (Figure 4K) was considerably lower in XJ25-Δ*glnR* than in XJ25, with a 16.3- and 7.0-fold increase in expression in XJ25+Glu and XJ25-Δ*glnR*+Glu, respectively, and a 0.37-fold decrease in expression in XJ25-Δ*glnR* compared with XJ25. Furthermore, the expression was considerably higher in XJ25-Δ*glnR*-36ek11-glnR and XJ25-ΔglnR-36ek11-glnR+Glu than in the corresponding strains XJ25-ΔglnR-36ek11 and XJ25-ΔglnR-36ek11+Glu, respectively. Collectively, the data suggest that glnR plays a vital role in improving the acid resistance of *L. plantarum* XJ25 by upregulating the transcription of gadB and glms1 and downregulating that of glnA and purQ.

### 3.5 Investigation of the interaction between glnR and glnA, gadB, glms1, and purQ via yeast one-hybrid assay

**FIGURE 5:**
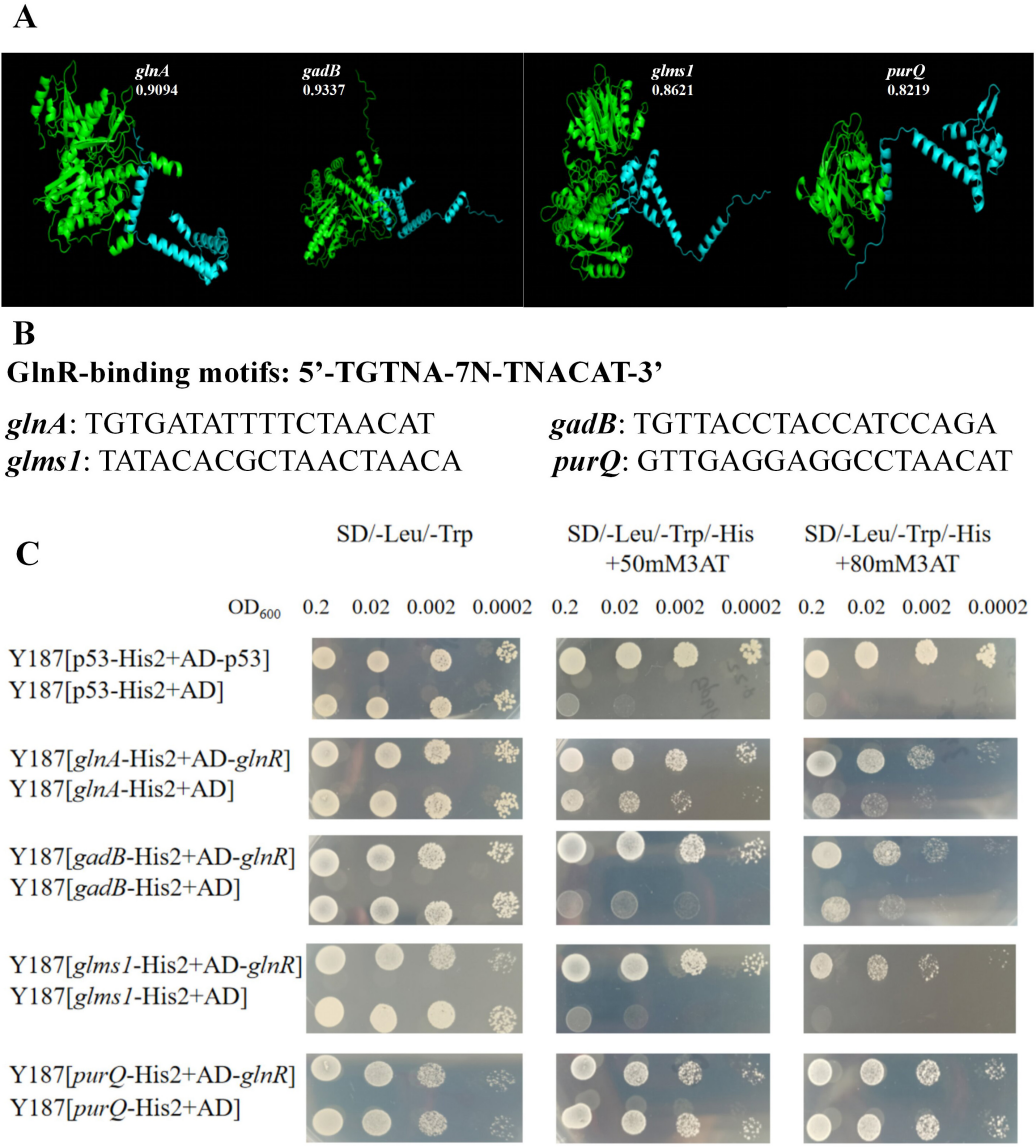
Yeast one-hybrid assay. **(A)** The confidence score of *glnR* and *glnA*, *gadB*, *glms1*, *purQ*. **(B)** The binding motif for *glnR* protein. **(C)** Yeast one-hybrid assay to display the interaction between *glnR* and *glnA*, *gadB*, *glms1*, *purQ*.

We performed homologous modeling to determine the three-dimensional structure of two genes and obtain interaction scores. When the confidence score is > 0.7, the possibility of two molecules interacting is high; when the confidence score is 0.5∼0.7, the two molecules may interact; and when the confidence score is < 0.5, the two molecules may not interact. Figure 5A illustrates that the confidence scores of glnR and glnA, gadB, glms1, and purQ are 0.9094, 0.9337, 0.8621, and 0.8219, respectively, suggesting that the possible interaction among these genes is higher. TGTNA-7N-TNACAT is the binding motif for glnR protein. glnA contains perfect 17-nt GlnR-binding motifs; in contrast, the regulatory regions of gadB, glms1, and purQ only contain sequences that have at least two mismatches with respect to the canonical motif (Figure 5B). To verify the binding of glnR to glnA, gadB, glms1, and purQ elements, we conducted the yeast one-hybrid assay to investigate the interaction between glnR and glnA, gadB, glms1, and purQ. Figure 5C illustrates the adequate growth of the yeast strains Y187 [p53-His2+AD-p53] and Y187 [p53-His2+AD] in SD/-Leu/-Trp medium; furthermore, the growth momentum of Y187 [p53-His2+AD-p53] was significantly better than that of Y187 [p53-His2+AD] in SD/-Leu/-Trp medium with 50 or 80 mM3AT. This suggests the interaction relationship between p53-His2 and AD-p53. Similarly, the growth momentum of Y187 [glnA-His2+AD-glnR], Y187 [gadB-His2+AD-glnR], and Y187 [glms1-His2+AD-glnR] was significantly better than that of Y187 [glnA-His2+AD], Y187 [gadB-His2+AD] and Y187 [glms1-His2+AD] in SD/-Leu/-Trp medium with 50 or 80 mM3AT, respectively. In particular, the yeast strains Y187 [purQ-His2+AD-glnR] and Y187 [purQ-His2+AD] exhibited similar growth momentums in all media, indicating that purQ was not affected by glnR. Collectively, these results suggest that glnR can bind to the promoter elements of glnA, gadB, and glms1 in yeast cells.

### 3.6 Changes in DAMs between XJ25 and XJ25-ΔglnR

**FIGURE 6:**
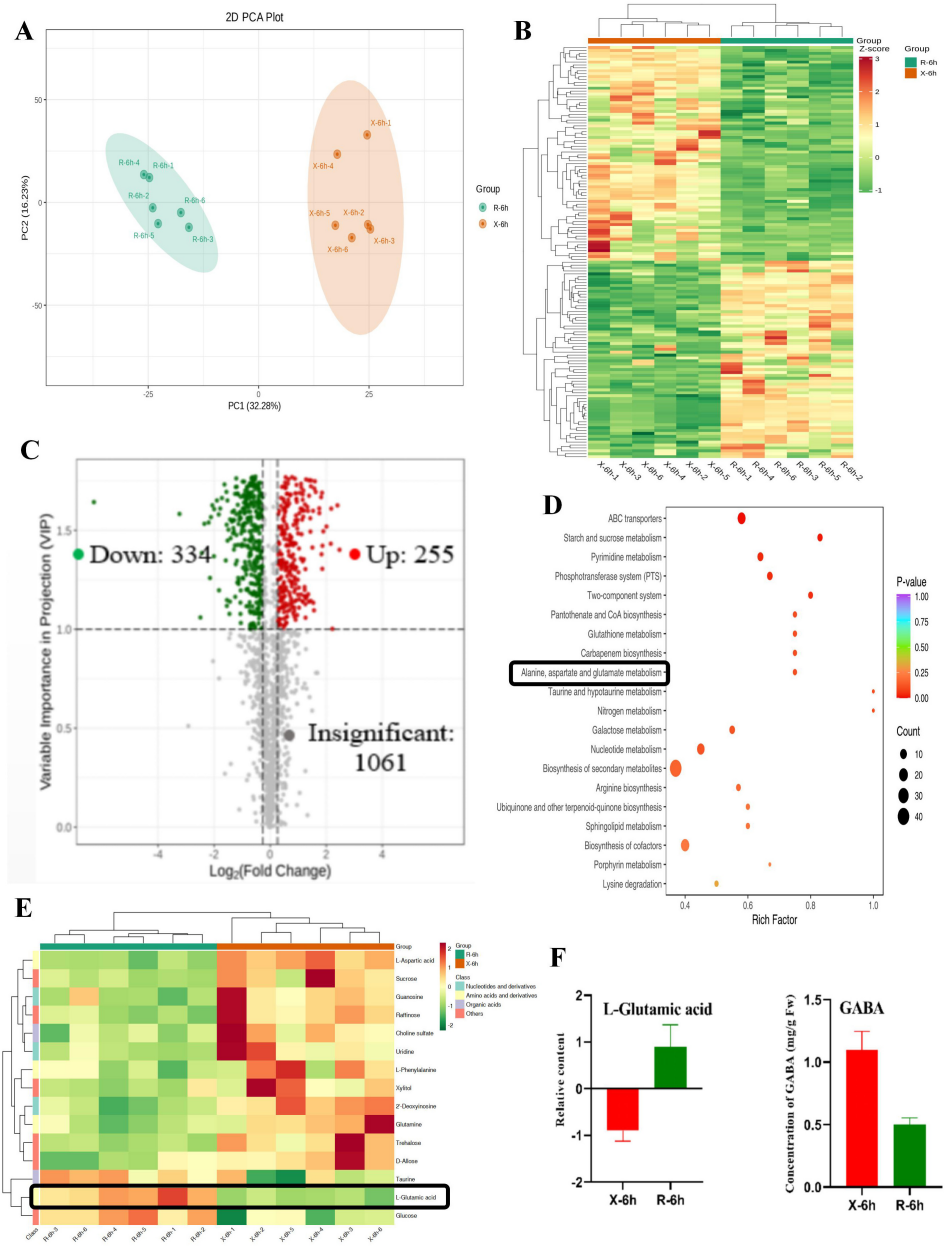
A nontargeted LC-MS metabolomics analysis of the metabolites in X J25 (X-6h) and XJ25-Δ*glnR* (R-6h). **(A)** PCA score plot of two test samples; **(B)** Heatmap of metabolites for X-6h vs. R-6h. The green color represents lower levels of metabolites, while the red color represents higher levels; **(C)** Volcano diagram of differentially accumulated metabolites (DAMs) identified from the one comparison (X-6h vs. R-6h). Green dots represent down-regulated metabolites, red dots represent up-regulated metabolites, and gray represents metabolites without significant differences. Numbers represent the identified differential metabolites of the pairwise comparisons; **(D)** Top 20 enriched KEGG pathways of differentially accumulated metabolites (DAMs) identified from **(C)**; **(E)** Differential metabolite clustering heatmap of KEGG pathway; **(F)** The concentration of L-Glutamic acid and GABA. Data are expressed as mean ± standard deviation (*n* = 6).

Using nontargeted LC-MS analysis, we identified 1,650 metabolites from XJ25 (X-6h) and XJ25-ΔglnR (R-6h). We performed PCA to better understand and analyze the dynamic alterations in the metabolites. Figure 6A illustrate that PC1 and PC2 explain 32.28% and 16.23% of the total variance, respectively. Therefore, PCA could effectively differentiate between X-6h and R-6h, indicating considerable changes in the metabolic profiles. Furthermore, the heatmap demonstrated a significantly different clustering (Figure 6B). Based on the orthogonal projections to latent structures differential analysis model, the differential metabolites were screened based on their VIP and FC values between both samples. Pairwise comparison revealed that the scores of R^2^Y and Q^2^ were > 0.9, suggesting that the model was appropriate (Supplementary Figure 2). The volcano plots in Figure 6C demonstrate the differences in the relative contents of the metabolites between X-6h and R-6h; in total, 589 DAMs were obtained, with 255 metabolites being upregulated and 334 being downregulated. KEGG enrichment analyses revealed that these metabolites were associated with the biosynthesis of secondary metabolites, ABC transporters, biosynthesis of cofactors, nucleotide metabolism, pyrimidine metabolism, and galactose metabolism, suggesting that these are the key metabolic pathways in response to GlnR (Figure 6D). Furthermore, as expected, alanine, aspartate, and glutamate metabolic pathways were detected. Five significantly enriched metabolic pathways were selected, and cluster analysis was performed on all metabolites in the pathways. L-Glutamic acid concentration was considerably higher in R-6h than in X-6h (Figures 6E, F), further confirming that among the core metabolites, L-glutamic acid is a metabolite that undergoes substantial changes in both samples. In contrast, GABA production was considerably lower in R-6h than in X-6h, suggesting that this pathway is associated with acid stress responses.

### 3.7 GlnR can directly regulate MLF in the model wine

**FIGURE 7:**
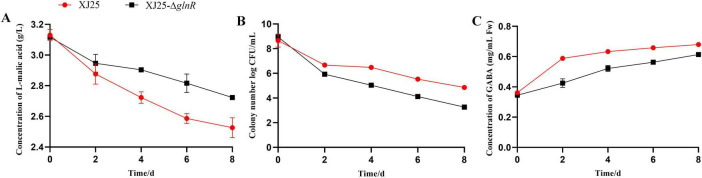
GlnR could directly regulate the process of MLF in the model wine. **(A)** L-malic acid utilization; **(B)** Dynamic changes of viable bacterial counts; **(C)** Dynamic changes of GABA. Data are expressed as mean ± standard deviation (*n* = 3).

The abovementioned data and analysis suggest that actively expressed *glnR* is essential for GABA production. To elucidate the importance of *glnR* in winemaking, XJ25 and XJ25-Δ*glnR* were cultured in model wine. Figure 7 illustrates the changes in L-malic acid concentration, viable bacterial counts, and GABA production during 8 days. The L-malic acid in both strains was not utilized after 8 days of fermentation. In XJ25, the L-malic acid concentration gradually decreased from 3.12 g/L to 2.53 g/L during MLF (Figure 7A); the residual L-malic acid concentration (2.72 g/L) was considerably higher in XJ25-Δ*glnR* than in XJ25. Furthermore, the viable bacterial counts of XJ25 were maintained at 10^5^ CFU/mL after MLF; the counts were higher than that of XJ25-Δ*glnR* (Figure 7B). Accordingly, in XJ25, GABA rapidly increases within the first 48 h and then moderately increases after 48 h (Figure 7C). Finally, GABA titer reached 0.679 mg/mL within 8 days of fermentation; this was considerably higher than that in XJ25-Δ*glnR* (0.614 mg/mL).

## 4 Discussion

The regulation of microbial acid tolerance is a complex process governed by various factors, especially transcription factors, which play a crucial role in enabling microorganisms to survive and thrive in acidic environments. This regulation is critical for both pathogenic and industrially relevant microorganisms. In the context of industrial applications, the engineering of transcription factors has been employed to enhance acid tolerance in various microorganisms. LAB survival plays a vital role in wine fermentation, the relevant characteristics of *L. plantarum*, including the ability to adapt well in high pH conditions, make it suitable to drive this process (Brizuela et al., 2019). Genomic context analysis (accession number: NZ_CP068448) has revealed that potential transcriptional regulator genes are annotated in *L. plantarum* XJ25, including *AcrR*, *LysR*, *Fnr*, *MarR*, *GntR*, *LacI*, *AraC*, *Rrf2*, *MerR*, and *XRE*. Transcriptional regulators can fine-tune the expression of associated genes in the metabolic network, adjust efficient microorganism growth and reproduction to cope with external stress environments, and thus play a vital role in defense responses to adversity (Khan et al., 2018; Vakulskas et al., 2015). However, information on the molecular mechanism underlying transcriptional factor-mediated acid resistance of bacteria remains limited. In the present study, we constructed the *glnR* mutant strains and discovered that the transcription factor *glnR* positively regulates the acid resistance of *L. plantarum* XJ25, this finding was not consistent with those reported by Gong et al. (2020), who observed that *glnR* negatively regulates the acid resistance of *L. brevis*. This apparent discrepancy may reflect differences in *Lactobacillus* species.

To investigate whether *glnR* causes a significant difference in metabolite concentrations in MLF-mediated winemaking, the wild-type and mutant strains were incubated in CDMm and model wine. Similar to the findings published by Chen et al. (2022), we observed that the L-malic acid consumption rate of the *glnR* knockout strains was slower rate than that of the wild-type strains under our culture conditions (Figures 3, 7). During MLF, one mole of malate is converted into one mole of lactate (Rizk et al., 2016). When malate concentration reaches < 0.3 g/L, MLF is considered complete (Marchante et al., 2019). L-malic acid can provide a sharp and strong taste sensation; however, high levels can result in a pungent taste (Robles et al., 2019). In the present study, L-malic acid was almost used up by all strains, except for strains XJ25-Δ*glnR* and XJ25-Δ*glnR*-36ek11 (Figure 3A). Presumably, *glnR* knockout strains the unsuccessful initiation of MLF or a longer fermentation time. Furthermore, the increased L-lactic acid concentration range was closely consistent with the decrease in L-malic acid concentration, consistent with the results of Li et al. (2018).

GlnR integrates intracellular nitrogen signals to modulate (induce or repress) the transcription of its target genes (Biswas et al., 2020). *GlnR* regulates many genes involved in nitrogen metabolism, including *gdhA*, *glnPQ*, *amtB*, *ureA*, *nirBD*, *glnK*, and *glnD* (Biswas et al., 2020; Amon et al., 2008; He et al., 2016). Qiu et al. (2022) have reported that *glnR* binds to the *Nir* promoter region and positively regulates its expression in *L. plantarum* WU14. Furthermore, Wang and Zhao (2009) have reported that GlnR positively regulates *nasA* transcription via specific binding in *S. coelicolor*. In *B. subtilis*, GlnR is a global regulator controlling four confirmed genes or operons, all of which are involved in nitrogen metabolism (Randazzo et al., 2017). In *L. brevis*, Gong et al. (2020) have reported that GlnR negatively regulates *glnA*, *gadB*, and glutamate-encoding gene (*gadC*) transcription. We observed that *glnR* regulates the expression of the genes (*glnA*, *gadB*, *purQ*, and *glms1*) involved in glutamate metabolism in the presence and absence of Glu. Moreover, the yeast one-hybrid assay was performed to further determine how *glnR* regulates the activity of *glnA*, *gadB*, *purQ*, and *glms1* promoters. The yeast one-hybrid assay is a classical method used to identify the specific transcription factors that transactivate a particular gene. The main aim of this assay can be summarized as determining which transcription factor specifically binds to given transcription factor-binding sites (Gaudinier et al., 2017). It is worth noting that the yeast one-hybrid assays support the ability of GlnR to bind the promoter regions of *glnA*, *gadB*, and *glms1*, but this heterologous system does not demonstrate promoter occupancy or regulatory dynamics in *L. plantarum*, suggesting *glnR* may upregulate *gadB* and *glms1* expression and downregulate *glnA* expression to enhance the acid resistance of *L. plantarum* XJ25.

Moreover, the GlnR-mediated acid metabolism regulation network is composed of multiple metabolites, *glnR* could modulate other metabolic pathways or physiological reactions, except for the acid resistance of the glutamate metabolite in *L. plantarum* XJ25. In the aspartate metabolic pathway, the content of aspartic acid in XJ25 was higher than that in the XJ25-Δ*glnR* mutant strain (Figures 4D, E). Aspartic acid metabolism can produce ammonia, which is used to maintain intracellular pH homeostasis in bacteria under highly acidic conditions (Hu et al., 2010). The study found that adding 0.04 g/L aspartic acid to MRS medium could improve the lyophilization survival rate of *L. plantarum* strains. This improvement is attributed to the increased peptidoglycan content in the cell wall with the adding of aspartic acid, which helps maintain cell integrity. Additionally, aspartic acid can elevate pH levels to reduce DNA damage (Chen et al., 2022).

**FIGURE 8:**
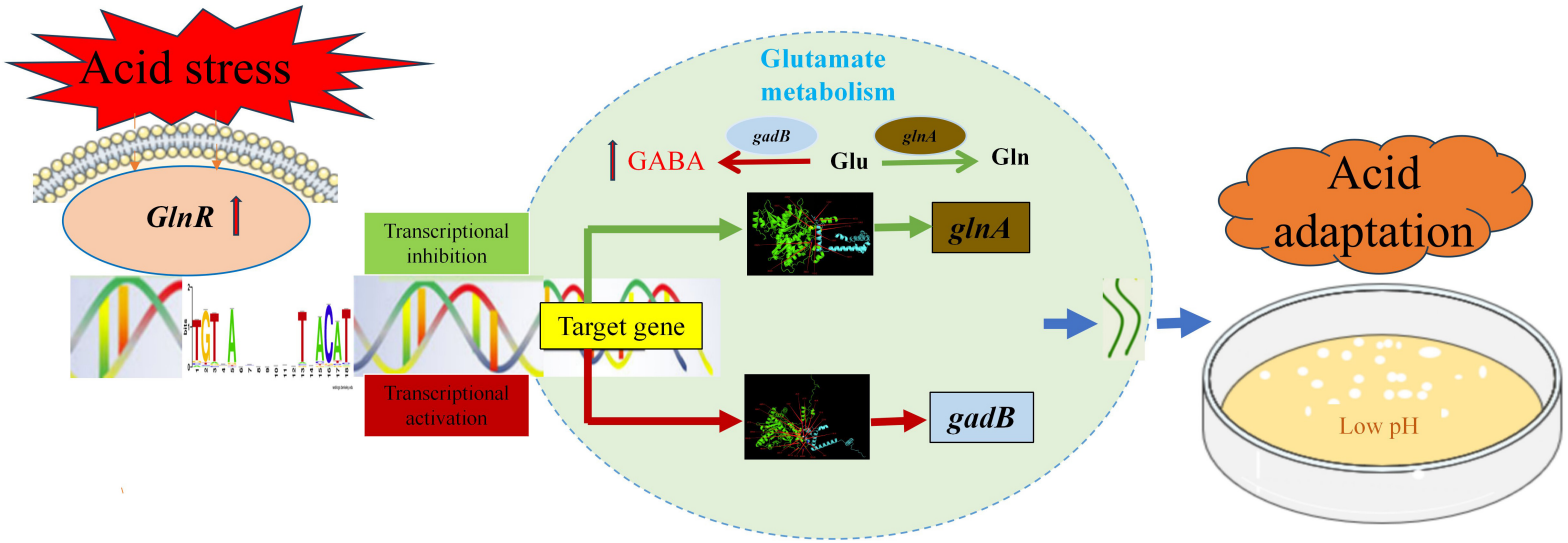
A proposed molecular model of *glnR* orchestrating glutamate metabolism to confer acid stress tolerance in XJ25. Red lines with arrowheads indicate positive regulation; Green lines with arrowheads indicate negative regulation. Glu, glutamate; Gln, glutamine; GABA, γ-aminobutyrate.

Figure 8 illustrates a proposed molecular model of *glnR* orchestrating glutamate metabolism to confer acid stress tolerance. The DNA sequence 5′-TGTNA-7N-TNACAT-3′ has been identified as the best *glnR*-binding motif (Kormelink et al., 2012). Under acid stress conditions, the relative expression of *glnR* increased (Figure 1A), subsequently controlling the expression of glutamate metabolism genes involved in GABA, glutamate, and glutamine to confer acid resistance. GABA is a ubiquitous nonprotein, four-carbon amino acid that plays a vital role in response to multiple abiotic and biotic stresses (Cataldo et al., 2020; Shelp et al., 2012). The application of exogenous GABA is associated with enhanced membrane stability (Shelp et al., 2012). XJ25-Δ*glnR* cells exhibited obvious wrinkling and collapse (Figure 2), these could be owing to a decrease in the conversion of glutamate to GABA.

Some LAB can tolerate acidic environments with the help of acid resistant genes. Genetic manipulation has helped identify the genes involved in acid resistance, including *argR*, *aspA*, *ilvE*, *cfa*, *hdcAPB*, *murG*, *gshA*, *gshB*, *trePP*, *pgmB*, *otsB*, *dnaK*, *shsp*, and *RecO* (Li et al., 2023; Guan and Liu, 2020). In *Escherichia coli*, *gadB* is the primary gene that helps convert glutamate to GABA; furthermore, it is the acid-resistant gene controlling acid resistance mechanisms (Ma et al., 2012). The acid resistance ability was slightly higher in in wild-type *L. brevis* strain than in the Δ*gadB* strain (Lyu et al., 2018). Although *glnR* can interact with *glnA*, *gadB*, and *glms1*, additional studies are warranted to determine how this interaction affects its acid resistance, facilitating our understanding of the mechanisms of *glnR* in acid resistance. In this study, we observed that *glnR* positively regulates the activity of the key enzyme *gadB* under acid stress, possibly activating the conversion process of glutamate to GABA. Therefore, additional studies on whether the gene *gadB* is the acid-resistant gene or not may be worthwhile.

## Data Availability

The data presented in this study are publicly available. The data can be found here: https://www.ncbi.nlm.nih.gov/nuccore/NZ_CP068448.1.

## Supplementary material

Supplementary Figure 1 Construction of glnR knockout mutant and expression mutant. **(A)** Electrophoresis profiles of glnR knockout mutant (lane 1 and 3), lane 2, 4–23: wild-type; lane 24: negative control; **(B)** sequencing validation of glnR knockout mutant; **(C)** electrophoresis profiles of glnR expression mutant, lane 1: XJ25; lane 2: XJ25-ΔglnR; lane 3: XJ25-ΔglnR-pMG36ek11; lane 4: XJ25-ΔglnR-pMG36ek11-glnR. Lane M: maker ladder 5000.

Supplementary Figure 2 OPLS-DA validation chart. The horizontal coordinate indicates the model R2Y, Q2 values.
