# Supplementary material for: GlnR positively affects the acid resistance of Lactiplantibacillus plantarum from wine by regulating glutamate metabolism
Source: Front Microbiol. 2026 Jan 22;16:1757806. doi: 10.3389/fmicb.2025.1757806 (PMC12872816; doi:10.3389/fmicb.2025.1757806)
Supplement: Supplementary file 1 [file Table_1.docx]

**Table S1.** Bacterial strains and plasmids.

| Strain | Genotype (description) | Source |
| --- | --- | --- |
| *E. Coli* DH5α | *Escherichia coli* | Laboratory |
| XJ25 | wild-type *L. plantarum* | Laboratory |
| pLCNICK | knockout plasmid | Song et al., 2017 |
| pLCNICK-Δ*glnR* | *glnR* knockout plasmid | This work |
| XJ25-Δ*glnR* | *glnR* knockout strain | This work |
| pMG36ek11 | Expression vector | Yang et al., 2022 |
| pMG36ek11-*glnR* | pMG36ek11 carrying *glnR* | This work |
| XJ25-Δ*glnR*-36ek11 | *glnR* knockout strain carrying pMG36ek11 | This work |
| XJ25-Δ*glnR*-36ek11-*glnR* | *glnR* knockout strain carrying pMG36ek11-*glnR* | This work |
| Y187 | Y187 yeast strain | Laboratory |
| pHis2 | pHis2 vector | Laboratory |
| pGADT7 | pGADT7 vector | Laboratory |
| AD-*glnR* | pGADT7 vector carrying *glnR* | This work |
| *glnA*-His2 | pHis2 vector carrying *glnA* | This work |
| *gadB*-His2 | pHis2 vector carrying *gadB* | This work |
| *glms1*-His2 | pHis2 vector carrying *glms1* | This work |
| *purQ*-His2 | pHis2 vector carrying *purQ* | This work |
| Y187[p53-His2+AD-p53] | Y187[p53-His2+pGADT7-p53] Yeast Strain | Laboratory |
| Y187[p53-His2+AD] | Y187[p53-His2+pGADT7] Yeast Strain | Laboratory |
| Y187[*glnA*-His2+AD-*glnR*] | Y187[*glnA*-His2+pGADT7-p53] Yeast Strain | This work |
| Y187[*glnA*-His2+AD] | Y187[*glnA*-His2+pGADT7] Yeast Strain | This work |
| Y187[*gadB*-His2+AD-*glnR*] | Y187[*gadB*-His2+pGADT7-p53] Yeast Strain | This work |
| Y187[*gadB*-His2+AD] | Y187[*gadB*-His2+pGADT7] Yeast Strain | This work |
| Y187[*glms1*-His2+AD-*glnR*] | Y187[*glms1*-His2+pGADT7-p53] Yeast Strain | This work |
| Y187[*glms1*-His2+AD] | Y187[*glms1*-His2+pGADT7] Yeast Strain | This work |
| Y187[*purQ*-His2+AD-*glnR*] | Y187[*purQ*-His2+pGADT7-p53] Yeast Strain | This work |
| Y187[*purQ*-His2+AD] | Y187[*purQ*-His2+pGADT7] Yeast Strain | This work |

**Table S2.** Primers used in this study.

| Primers | Sequence (5′-3′) |
| --- | --- |
| *glnR*-up-1 | ctttttctaaactagggcccAGCAGTCGGTAAGACGG |
| *glnR*-up-2 | GCAAACATCTTACATAACGACCCTCCTCTTTAATAC |
| *glnR*-down-1 | GGAGGGTCGTTATGTAAGATGTTTGCATTCGTTAGTC |
| *glnR*-down-2 | ccgagtcggtgctttttttGTTAGATGCTGACCAGGCC |
| sgRNA-1 | aaaaaaagcaccgactcgg |
| *glnR*-sgRNA-2 | ggatgatatcacctctagaCGACAAGGGCCCCAATTATCgttttagagctagaaatagc |
| *glnR*-ha-1 | CGACTGGATCAATCTATTGGAAC |
| *glnR-*hp*-2* | CCGTGCACTTGGAATCC |
| *glnR*-in-1 | GAAGGAAAAGGAACTCCGTCG |
| *glnR*-in-2 | GTGTGCCGGATAATTGGGG |
| pLCNICK-test-1 | aaaagggatagtaattcattcctgg |
| pLCNICK-test-2 | tgcgagttgaccgtggg |
| P11-2 | ttcaaaattcctccgaatAGCAACATTATATCATAGTATGTCCATTCTGT |
| pMG36e-express-3 | aaaggcgacggtacaaccggtactagcgac |
| *glnR*w-express-1 | aggtaaaaaaaTattcggaggaattttgaaATGAAGGAAAAGGAACTCCGTCGCTCGTT |
| *glnR*w-express-2 | aaggttcaaaatattaaattttaccggtcaTTAGTGTGCCGGATAATTGGGGCCCTTGT |
| pMG36e-test-1 | gcacggtcgatcttctatat |
| pMG36e-test-2 | tcgcaacagaaccgtttcta |
| *gabD*-1 | GCTGATTACTATGCTGATCAC |
| *gabD*-2 | GATTCCAGCCATATTGATGC |
| *gdh*-1 | AATGTTAGCACCACTCAG |
| *gdh*-2 | GATAATCGCCTCAAGCAA |
| *glnR*-1 | CGCTACTATGAAGAGCAAG |
| *glnR*-2 | GATTCCAGCCATATTGATGC |
| *gadB-1* | ATTGGTATCGTCGGTATC |
| *gadB-2* | CTGATGATTGTAGTGAGTAAC |
| *purQ*-1 | CGTCATAACCTGCTAATG |
| *purQ*-2 | CCTGGTTCTAATTGTGATC |
| *glnA*-1 | TGCCTAAGCCGTTAAACG |
| *glnA*-2 | AAGTGATAAGCGTCTGAT |
| *glms1*-1 | GCATACAACCAACCATTA |
| *glms1*-2 | CACATTCAATAACACTTCAC |
| *purF*-1 | AACTGACCGACGACTAAT |
| *purF*-2 | TTGCTTACTTACTACTAACGAAT |
| *lp_0433*-1 | CGTTACAATTACTCAGCAAG |
| *lp_0433*-2 | CGATCGACGACTAACAT |
| *carB*-1 | TGACCAACCTGATAATCC |
| *carB*-1 | GGTGTTACTGCTAATAATGT |
| 16SrRNA-1 | GCAACGAGCGCAACCC |
| 16SrRNA-2 | GACGGGCGGTGTGTAC |
| AD-F | TAATACGACTCACTATAGGGCG |
| AD-R | TACTGAAAAACCCCGCAAGTTC |
| pHis2-1 | TTCGCTATTACGCCAGCTG |
| pHis2-2 | GTTTATCTTGCCTGCTCATT |
| AD-*glnR*-express-1 | ccatacgacgtaccagattacgctcatatgATGAAGGAAAAGGAACTCCGTCGCTCGTT |
| AD-*glnR*-express-2 | attcatctgcagctcgagctcgatggatccTTAGTGTGCCGGATAATTGGGGCCCTTGT |
| His-*glnA*-express-1 | atacgactcatcatagggcgaattcTTGACCAACGGCTAGCTAGT |
| His-*glnA*-express-2 | gatcgattcgcgaacgcgtgagctcCTTCATAACGACCCTCCTCT |
| His-*gadB*-express-1 | atacgactcatcatagggcgaattcTTGGGGCAAAGATCCTCACT |
| His-*gadB*-express-2 | gatcgattcgcgaacgcgtgagctcCGAGTATAGCACGAGGTGAT |
| His-*glms1*-express-1 | atacgactcatcatagggcgaattcGACGACTTACCCACAAAAGT |
| His-*glms1*-express-2 | gatcgattcgcgaacgcgtgagctcGTACCAAGACGCGACCATCA |
| His-*purQ*-express-1 | atacgactcatcatagggcgaattcGAAGTATTGACGCGGCTAGCAA |
| His-*purQ*-express-2 | gatcgattcgcgaacgcgtgagctcCGACATTCGTATATCCAAGTCG |
