# Supplementary figures and images for: GlnR positively affects the acid resistance of Lactiplantibacillus plantarum from wine by regulating glutamate metabolism
Source: Front Microbiol. 2026 Jan 22;16:1757806. doi: 10.3389/fmicb.2025.1757806 (PMC12872816; doi:10.3389/fmicb.2025.1757806)

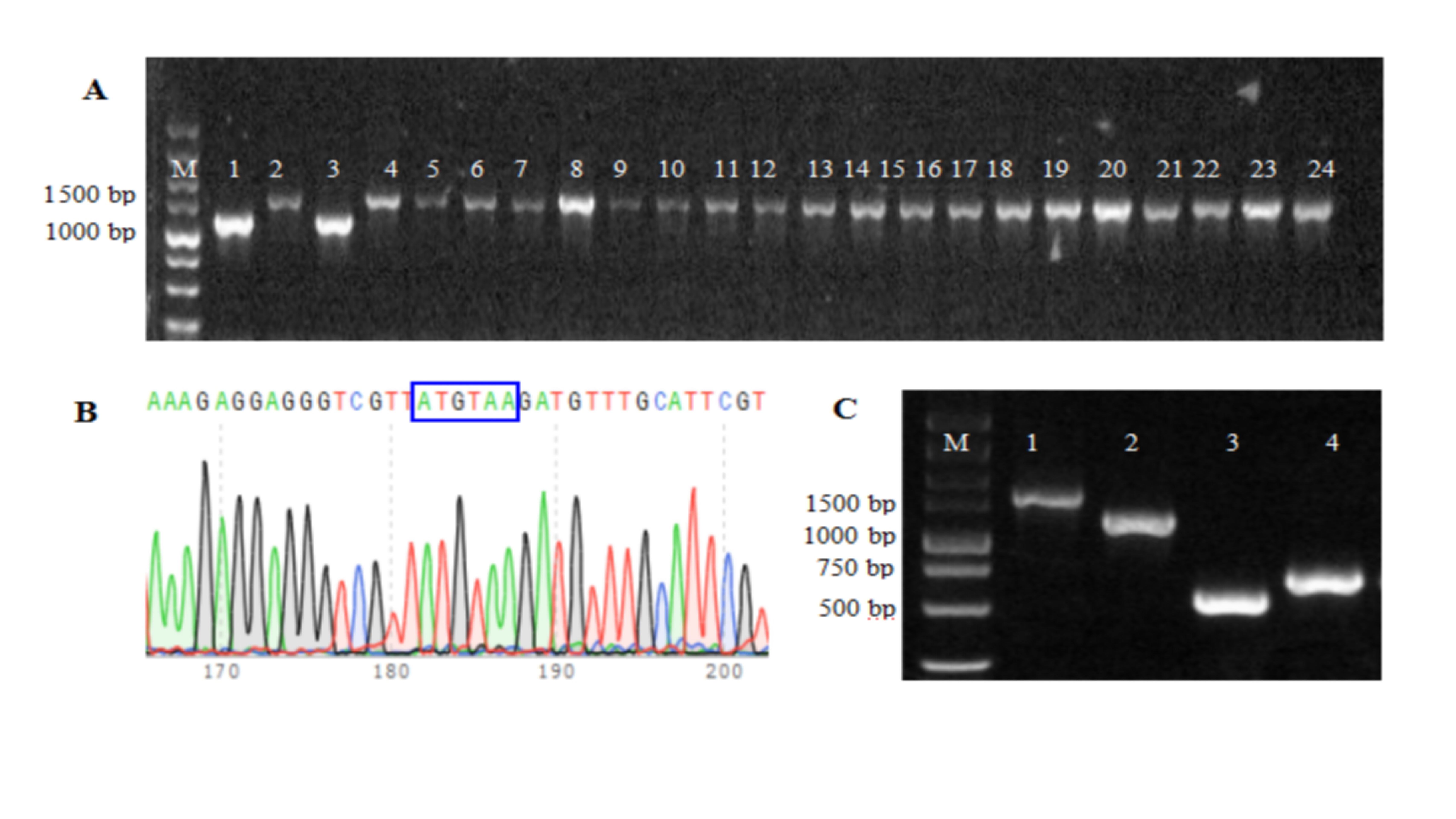

Supplement: Supplementary Figure 1 — Construction of glnR knockout mutant and expression mutant. (A) Electrophoresis profiles of glnR knockout mutant (lane 1 and 3), lane 2, 4–23: wild-type; lane 24: negative control; (B) sequencing validation of glnR knockout mutant; (C) electrophoresis profiles of glnR expression mutant, lane 1: XJ25; lane 2: XJ25-ΔglnR; lane 3: XJ25-ΔglnR-pMG36ek11; lane 4: XJ25-ΔglnR-pMG36ek11-glnR. Lane M: maker ladder 5000. [file Image_1.jpeg]

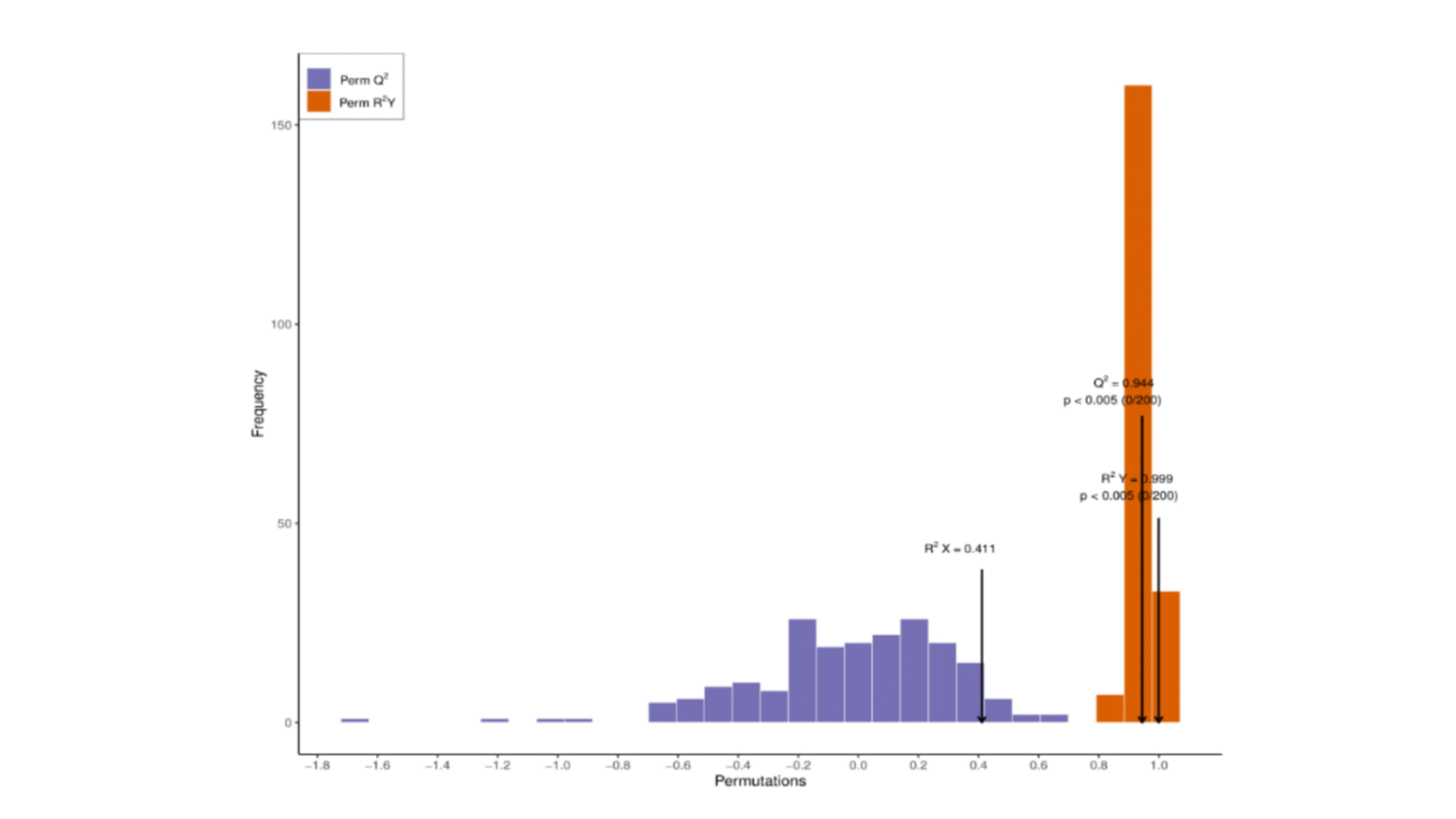

Supplement: Supplementary Figure 2 — OPLS-DA validation chart. The horizontal coordinate indicates the model R2Y, Q2 values. [file Image_2.jpeg]
